# Early diagnosis of celiac disease in IgA deficient children: contribution of a point-of-care test

**DOI:** 10.1186/1471-230X-14-186

**Published:** 2014-11-06

**Authors:** Francoise Bienvenu, Silvia I Anghel, Cécile Besson Duvanel, Julien Guillemaud, Lorna Garnier, Florian Renosi, Alain Lachaux, Jacques Bienvenu

**Affiliations:** Lyon-Sud Hospital-Immunology Laboratory, 69495 Pierre-Bénite Lyon, France; Augurix SA, Monthey, CH-1870 Switzerland; Paediatric Hospital-Gastroenterology-Hepatology-Nutrition Department, Hospices Civils de Lyon, Lyon, 69677 Bron, France

**Keywords:** Celiac disease, IgA deficiency, Deamidated gliadin peptides, Point-of-care test

## Abstract

**Background:**

The serological diagnosis of celiac disease (CD) often relies on the presence of anti-tissue transglutaminase (tTG) IgA autoantibodies. Patients suffering from selective IgA deficiency (IgAD) are often not aware of their IgA deficiency and are tested as CD negative, delaying considerably the diagnosis. The detection of IgG against deamidated gliadin peptides (DGP) has high specificity and better sensitivity than IgG anti-tTG. A multi-analytic lateral-flow immunochromatographic assay (CD-LFIA) based on the detection of IgA and IgG anti-DGP and total IgA was shown to have a good diagnostic accuracy for CD. The aim of this study was to evaluate the clinical accuracy of its use in children suffering from IgAD.

**Methods:**

45 IgAD children ranging from 1.1 to 17.4 years and suspected of CD or having high CD risk factors were referred from outpatient clinics located in the area of Rhone-Alpes (France) to the Hospices Civils de Lyon, Paediatric Hospital-Gastroenterology-Hepatology- Nutrition Department for further CD investigations. The CD investigations, including the sample collection, were performed within the Paediatric Hospital-Gastroenterology-Hepatology- Nutrition Department, and the serological testing was performed at the Lyon-Sud Hospital-Immunology Laboratory. The diagnosis of CD was based on IgG anti-tTG serology, biopsy results and patient follow-up. The serum samples were retrospectively tested on the CD-LFIA test.

**Results:**

A total of eight (8) patients were diagnosed as new CD. All were correctly identified by the CD-LFIA. The test yielded four (4) false positive results. Two patients with positive IgG anti-tTG were negative on CD-LFIA, but were classified as CD negative based on biopsy results and patient follow-up. The remaining 33 patients were found negative by both methods. The specificity and sensitivity of CD-LFIA was of 89.2% [74.6-97.0] and of 100% [63.1-100] respectively. The negative predictive value (NPV) was of 100% [89.4-100], and the Likelihood Ratio for Negative Test (LR-) was of 0 [0.0-0.91].

**Conclusions:**

CD-LFIA is a useful, non-invasive and rapid tool to rule out CD in primary care paediatric patients having CD-related symptoms and IgAD. Patients having a positive CD-LFIA result could be then readily directed to secondary care setting for further evaluation by standard serology and biopsy.

## Background

Selective IgA deficiency (IgAD) is a common immunodeficiency occurring in Caucasians with a prevalence rising up to 1:600 [1, 2]. IgAD is characterized by total IgA serum levels below 0.06 g/L and normal levels of IgM and IgG [3]. Although the majority of IgAD individuals are asymptomatic, IgAD is associated with autoimmune disorders such as celiac disease (CD) [4]. Its frequency is raised and estimated to be about 1:40 among patients suffering from CD [5–7].

The European Society for Paediatric Gastroenterology Hepatology and Nutrition (ESPGHAN) strongly recommends testing for the presence of IgA autoantibodies against tissue transglutaminase (tTG) as the initial step for CD diagnosis [5, 8, 9]. Moreover, in cases where the total IgA status is unknown, the guidelines strongly recommend IgA measurement [8–10]. In case of IgAD, a positive IgG anti-Endomysium (EMA) [8], anti- tTG or anti- deamidated gliadin peptides (DGP) antibodies is considered as diagnostically relevant [8–10].

Unfortunately, investigation of the total IgA levels is frequently neglected, and patients suffering from IgAD are often not aware of their IgA deficiency and may therefore be tested as CD negative, delaying considerably the diagnosis [3, 11]. Recently, it has been demonstrated that the detection of IgG against DGP has a high specificity and a better sensitivity than IgG anti-tTG [12–16]. Therefore, their use was suggested as an alternative for the diagnosis of CD in IgAD patients [3].

A multi-analytic lateral-flow immunochromatographic assay (CD-LFIA) based on the rapid detection of both IgA and IgG anti-DGP and total IgA has previously been shown to have a good diagnostic accuracy to rule out CD in high-risk paediatric and adult populations [17, 18].

The aim of the present study was to evaluate its use as a tool to rule out CD in children suffering from IgAD.

## Methods

### Patients

Paediatric patients suspected of CD or having high CD risk factors were referred from outpatient clinics located in the area of Rhone-Alpes (France) to the Hospices Civils de Lyon, Paediatric Hospital-Gastroenterology-Hepatology- Nutrition Department for further CD investigations. The CD investigations, including the sample collection, were performed within the Paediatric Hospital-Gastroenterology-Hepatology- Nutrition Department, and the serological testing was performed at the Lyon-Sud Hospital-Immunology Laboratory. From 2001 to 2012, 45 paediatric patients were selectively diagnosed with IgAD with total IgA levels below 0.06 g/L. The study stems from collaboration between the Paediatric Hospital-Gastroenterology-Hepatology- Nutrition Department and the Lyon-Sud Hospital-Immunology Laboratory. Ethical approval was obtained from the Ethical Committee of Hospices Civils de Lyon for the retrospective use of the samples collected within the Paediatric Hospital-Gastroenterology-Hepatology- Nutrition Department and analyzed within the Lyon-Sud Hospital-Immunology Laboratory. The samples were anonymized, and information was collected on the CD related symptoms and/or high risk factors, on the serological investigation, biopsy results, and finally on the patient follow-up when available. From the 45 IgAD paediatric patients, 17 were tested because of clinical suspicion of CD (diarrhea, anemia, and failure to thrive), 18 because of risk factors for CD (autoimmune diseases among which 14 suffer from type 1 diabetes), and finally ten (10) patients were not clinically documented. To our knowledge, none of the patients included in this study were diagnosed with another small intestinal disorder at the time of CD diagnosis.

### Diagnostic methods

Total IgA was measured using the BNII nephelometer (Siemens) according to the manufacturer’s protocol. IgAD was diagnosed when total IgA levels were below 0.06 g/L. For the study population, normal values ranged between 0.12 g/L and 2.03 g/L, depending on the patient’s age.

The CD diagnosis was made by the gastroenterologist, and was based on results of serologic enzyme-linked immunosorbent assay (ELISA) IgG anti-tTG test (see below), small intestine mucosal biopsy examination and on patient follow-up when available.

IgG anti-tTG levels were measured by the ELISA test Celikey^®^ from Thermo Fisher (Uppsala, Sweden). Concentrations >5 U/mL were considered as positive.

In case of discrepancy between IgG anti-tTG and CD-LFIA, IgG anti-DGP levels were measured by the ELISA test Varelisa^®^ Gliadin Antibodies from Thermo Fisher (Uppsala, Sweden). Concentrations >10 U/mL were considered as positive.

At endoscopy, 4–6 duodenal biopsies were taken from patients having a positive IgG anti-tTG serology [8]. The duodenal biopsies were analyzed by an experienced histopathologist, who assessed the following pathologic features of CD: villous atrophy, crypt hyperplasia, increased intraepithelial lymphocytes, and chronic inflammation in the lamina propria. The diagnosis of CD was subsequently confirmed according to the modified Oberhuber-Marsh classification [19].

### CD-LFIA test

The serum samples were tested by CD-LFIA test (Simtomax^®^ Augurix, Switzerland) according to the manufacturer’s instructions. The CD-LFIA point-of-care device detects simultaneously both human IgA and IgG anti-DGP, as well as total IgA in 10 to 15 minutes [17, 18]. Briefly, the test was read as CD positive and IgAD when both the control line and the line corresponding to anti-DGP detection could be seen. Each sample was tested on one device by two independent user-operators blinded to the subject’s history and laboratory findings. Prior to the clinical study, each user-operator had completed a one-day course on the use of CD-LFIA.

### Statistical analysis

The laboratory ELISA IgG anti-tTG, biopsy results, and patient follow-up were used as “standard diagnosis” for comparative analyses to evaluate the testing features of CD-LFIA.

The CD-LFIA test’s sensitivity, specificity, diagnostic accuracy, and positive and negative likelihood ratios (LR+, LR-) and their corresponding 95% confidence intervals (CIs) were calculated using the STATA software (version 11; College Station, TX, USA).

## Results

### Patient’s characteristics

The diagnostic performance of CD-LFIA was retrospectively tested on serum samples from 45 paediatric patients diagnosed as IgAD, and initially tested by ELISA and biopsy (when requested) because of clinical suspicion of CD or risk factors for CD. Ten (10) patients were not clinically documented.

The age of the population ranged from 1.1 to 17.4 years, with a mean and median age of 8.2 and 8.4 years, respectively, with 40% of females (n = 18), and 60% of males (n = 27). 50% of females were initially investigated because of CD-related symptoms, whereas 28% of them were at high-risk of CD (diagnosed with autoimmune diseases). In contrast, the majority of the male population was suffering from autoimmune disease (48%, with a clear overrepresentation of type 1 diabetes, n = 11), whereas 30% were suffering initially from CD-related symptoms (Table 1).

**Table 1:** **Characteristics of patients participating to the study**

Gender	Total population	Population of CD related symptoms	Population having risk factors (Autoimmune disease)	Population having unknown clinical symptoms	Age range (years)	Mean age (years)	Median age (years)
Females	18	9	5	4	1.3 to 17.4	8.4	8.8
Males	27	8	13	6	1.1 to 16.2	8.1	7.3

### Overall agreement between CD-LFIA and the “standard diagnosis”

**Figure 1:**
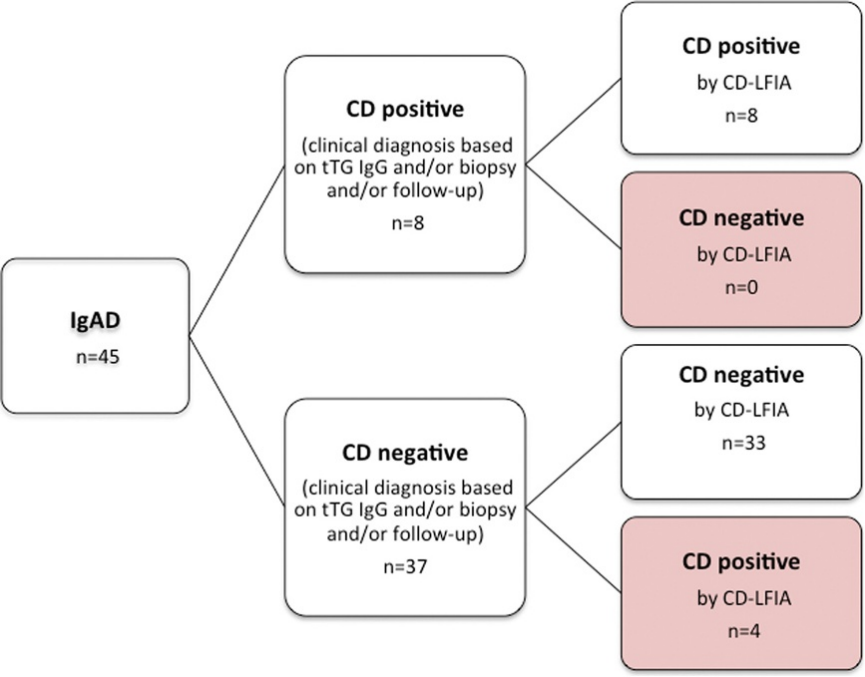
**Histogram comparing standard diagnosis by IgG anti-tTG ELISA assay and/or biopsy and/or follow-up to CD-LFIA test results.**

Based on the “standard diagnosis” (IgG anti-tTG positive serology, biopsy result and patient follow-up), eight (8) new cases of CD were found, all were correctly identified by CD-LFIA (Figure 1). Their histology revealed subtotal (n = 1; Marsh 3b) or complete villous atrophy (n = 7; Marsh 3c).

From these eight cases, five (5) were among females and were all initially tested because of CD related symptoms, particularly diarrhea, whereas among the three (3) newly identified celiac positive male patients, one had CD related symptoms, and two (2) were suffering from type 1 diabetes. The age of the newly diagnosed CD ranged from 1.5 to 17.4 years.Thirty-seven patients were diagnosed as celiac negative. Among them, 33 were correctly identified by CD-LFIA. Four (4) patients were identified as positive on CD-LFIA. The IgG anti-DGP levels of these four (4) patients were further measured by ELISA and among them, two (2) had positive levels of IgG anti-DGP. One of these patients underwent duodenal biopsy one year later, and the result showed a negative histology, and was therefore considered as CD negative. The second patient (2.1 years old), showed IgG anti-tTG levels near the cut-off (3.4 U/ml instead of 5 U/ml) and positive levels of IgG anti-DGP (16 U/ml). Unfortunately, this patient had no follow-up and no biopsy was conducted. Both patients were further considered as false positive (Figure 1).Among the celiac negative patients, two (2) patients were weakly positive on the ELISA IgG anti-tTG (5 and 9.9 U/ml) and negative on CD-LFIA and ELISA IgG anti-DGP. Based on their medical follow-up and their normal biopsies they were considered as CD negative. Thus, no false negative results were detected using CD-LFIA (Figure 1).

### Diagnosis in patients suffering from other autoimmune disease

Among the 45 paediatric patients, 18 were suffering of other autoimmune diseases, among which 15 were suffering of type 1 diabetes. Fourteen (14) of them had concordant results between ELISA IgG anti-tTG, ELISA IgG anti-DGP and CD-LFIA.

Among the four remaining patients, two patients (6 and 15.6 years males) had positive ELISA IgG anti-tTG results (5 and 9.9 U/ml) but were diagnosed as CD negative based on their biopsy and follow-up. Both patients had negative CD-LFIA and ELISA IgG anti-DGP results. A 5.6 years old male suffering from type 1 diabetes was found negative on both ELISA IgG anti-tTG and anti-DGP but was positive on CD-LFIA. Finally, an 11.2 years old female diagnosed as CD negative based on ELISA IgG anti-tTG result had a negative CD-LFIA result and a positive ELISA IgG anti-DGP result (43 U/ml). Unfortunately, no biopsy and further follow-up was conducted.

### Evaluation of the diagnostic performance of CD-LFIA

**Figure 2:**
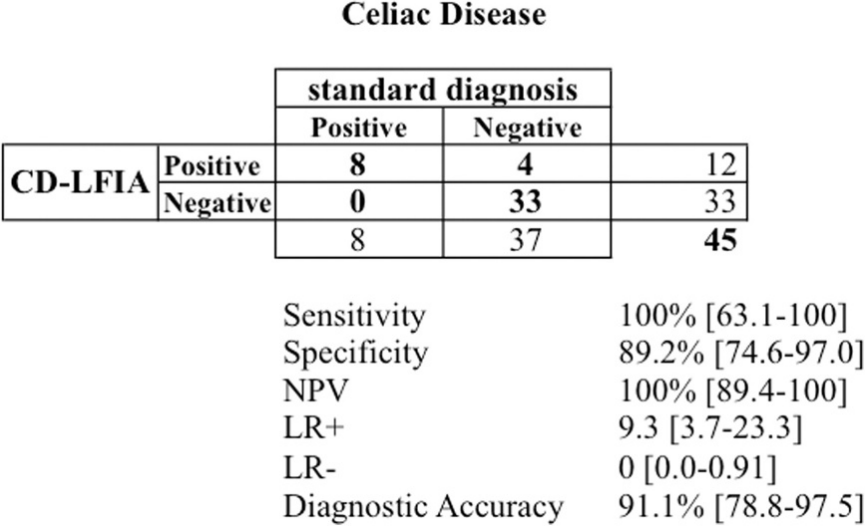
**Diagnostic performance of CD-LFIA [with 95% CI]**.

These results yield a sensitivity for CD-LFIA device of 100% [63.1-100], and a specificity of 89.2% [74.6-97.0] (Figure 2). The Negative Predictive Value (NPV), which takes into account false negative results, was of 100% [89.4-100] and the Likelihood Ratio for Negative Test (LR-) was of 0 [0.0-0.91] (Figure 2). The overall diagnostic accuracy was of 91.1% [78.8-97.5].

## Discussion

The diagnosis of CD is a challenge for many physicians. Unfortunately, up to 75% of CD remains undiagnosed, and the time between the onset of symptoms and diagnosis is often too long [20, 21]. It is thought that the use of a highly sensitive rapid point- of- care test, performed in primary care centers, may help physicians to rule out CD and to select patients on which more invasive diagnostic tests are required [22–24]. This strategy could allow a better and more reactive patient support, decreasing the time to diagnosis while increasing the patient quality of life.

According to the last ESPGHAN guidelines, the initial testing of patients suspected of CD should be the serological identification of IgA autoantibodies against tTG, followed by biopsy in case of a positive serology [8]. In case of serology/biopsy disagreement, measurement of total IgA levels and IgG anti-tTG or anti-DGP as well as HLA-DQ genotyping are recommended. However, based on epidemiological studies, it is known that IgAD occurs with a higher frequency in CD positive patients [2, 4–7]. Regardless the fact that ESPGHAN recommends the measurement of total IgA levels [8] their determination is often neglected, which obviously delays even more the time to diagnosis of CD in patients suffering from IgA deficiency. Therefore, there is a clear need for reliable and easy to perform tests in primary care centers, that could detect CD regardless of the total IgA status of patient.

As mentioned above, the initial standard serological test is based on IgA anti-tTG antibodies. In case of IgAD, one can think of using IgG anti-tTG or anti-EMA antibodies. However, several clinical studies showed that IgG anti-tTG antibodies are not as sensitive and specific as the IgA anti-tTG antibodies, and the use of EMA assay in daily practice is limited by high costs and subjective interpretations [25]. In contrast, several studies showed that the detection of IgG against DGP has a high specificity and a better sensitivity than IgG anti-tTG [12–16, 26] and this seems to be also true in case of IgA deficiency [3, 27, 28].

A multi-analytic lateral-flow immunochromatographic assay (CD-LFIA) based on the rapid detection of both IgA and IgG anti-DGP and total IgA was previously shown to have a good diagnostic accuracy to rule out CD in high-risk paediatric and adult populations [17, 18]. Since the test detects IgA and IgG antibodies against DGP and total IgA levels and it has a very good sensitivity and NPV value, it seems to us that the test could facilitate the management of paediatric patients, including undiagnosed IgAD, consulting in primary care centers for CD-related symptoms. Therefore, the aim of the present study was to evaluate its use as a tool to rule out CD in children suffering from IgAD.

Within the 45 paediatric IgAD patients included in the study, eight (8) were newly identified CDs, all of them were correctly identified by CD-LFIA. From the remaining 37 patients, which were considered as CD negative, 33 were correctly identified by CD-LFIA and four (4) were considered as false positive. Among them, one patient underwent duodenal biopsy and was subsequently diagnosed CD negative. A second patient (2.1 years old) had positive levels of IgG anti-DGP by ELISA. Although the recent ESPGHAN guidelines recommend the use of antibodies against DGP in children younger than 2, and several papers showed that antibodies against DGP outperform EMA and tTG antibodies in children under 2 years of age, the patient had no further follow-up and no endoscopic examination [8, 9, 13, 28, 29]. From the 33 CD negative patients, two (2) had a positive IgG anti-tTG serology but were diagnosed as CD negative based on their biopsy results and follow-up. Both patients were correctly diagnosed by CD-LFIA, and therefore CD-LFIA did not yield false negative results.

Limitations that apply to our study are foremost its retrospective nature in a high pretest population. The decision of conducting the study retrospectively in a secondary care setting was principally guided by the low prevalence of IgAD that could eventually increase the length of the study. Clinical symptoms were missing for ten (10) of the 45 patients included in this study. However, diagnostic agreement was found between the serologic IgA anti-tTG and CD-LFIA for all these patients, all being CD negatives.

## Conclusion

With a sensitivity of 100% [63.1-100], a NPV of 100% [89.4-100] and a LR- of 0 [0.0-0.91], we showed here that CD-LFIA is a useful, non-invasive and rapid tool to rule out CD in primary care paediatric patients having CD-related symptoms and IgAD. By detecting in a single test, both IgA/IgG anti-DGP and total IgA, CD-LFIA appears to be well adapted to the recommendations concerning the systematic investigation of IgA levels in patients screened for CD [8–10]. Patients having a positive CD-LFIA result could be then readily directed to secondary care setting for further evaluation by standard serology and biopsy.
